# Spatial transcriptomes and microbiota reveal immune mechanism that respond to pathogen infection in the posterior intestine of *Sebastes schlegelii*

**DOI:** 10.1098/rsob.220302

**Published:** 2023-02-22

**Authors:** Min Cao, Ting Xue, Huijun Huo, Xiaoyan Zhang, Ning Ning Wang, Xu Yan, Chao Li

**Affiliations:** School of Marine Science and Engineering, Qingdao Agricultural University, Qingdao 266109, People's Republic of China

**Keywords:** spatial transcriptome, posterior intestine, microbiota composition, *Edwardsiella piscicida*, *Sebastes schlegelii*

## Abstract

The intestine is a site of immune cell priming at birth. Therefore, spatial transcriptomes were performed to define how the transcriptomic landscape was spatially organized in the posterior intestine of *Sebastes schlegelii* following *Edwardsiella piscicida* infection. In the healthy condition, we identified a previously unappreciated molecular regionalization of the posterior intestine. Following bacterial infection, most immune-related genes were identified in mucosa layer. Moreover, investigation of immune-related genes and genes in immune-related KEGG pathways based on spatial transcriptomes shed light on which sections of these genes are in the posterior intestine. Meanwhile, the high expression of genes related to regeneration also indicated that the posterior intestine was responding to the invasion of pathogens by constantly proliferating new cells. In addition, the increasing microbiota communities indicated that these bacteria maintained posterior intestine integrity and shaped the mucosal immune system. Taken together, spatial transcriptomes and microbiota compositions have significant implications for understanding the immune mechanism that responds to *E. piscicida* infection in the posterior intestine of *S. schlegelii*, which also provides a theoretical basis for the spatial distribution of immune genes and changes in bacterial flora in other teleosts in the process of resisting pathogens.

## Graphical abstract

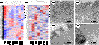

## 1. Introduction

The intestine performs the critical function of nutrient uptake and utilization, supplying the energetic and metabolic demands of most organisms [1], and is also a site of immune cell priming at birth [2]. Owing to constant exposure to antigens and immunomodulators from diet and commensal microbiota, the intestine also serves as a port of entry for many pathogens. Therefore, intestinal immune processes were increasingly involved in controlling disease development elsewhere in the organism [3]. Usually, the intestine is formed by the mucosa, the underlying lamina propria, the muscularis mucosa and serosa [4]. Among these, the epithelial surface in the mucosa plays a critical role in host protection by producing a diverse repertoire of antimicrobial proteins that directly kill or hinder the growth of microorganisms [5]. When intestinal barrier injury occurs, immune cells are recruited or expanded *in situ* to protect the host from invading pathogens and to orchestrate the healing process by providing resolving signals [6].

In addition, the intestine is also a habitat for microorganisms that play protective roles in the process of pathogen infection, by expressing immune genes or secreting mucus to resist the invasion of pathogenic bacteria. It has been reported that the dense resident microbial community in the intestine co-evolved with the host and was essential for many host physiological processes, including enhancement of the intestinal epithelial barrier, development of the immune system and acquisition of nutrients. The mechanisms that regulate the ability of the microbiota to restrain pathogen growth are complex and include competitive metabolic interactions, localization to intestinal niches and induction of host immune responses [7]. Such a vital role offers a unique opportunity to investigate the barrier and fundamental function of the intestine in immunity.

As the organ with the largest surface area contact with the external environment, the fish intestine is easily affected by physiological damage [8]. Damage to the intestinal barrier leads to increased intestinal permeability. Therefore, pathogens and harmful substances in the intestine will enter the blood system and be transmitted to other organs and tissues in the body, triggering a systemic pathological response, which will lead to a major impact on the fish health [9,10]. Most of the fish intestine is divided into three portions according to thickness of the wall, length of mucosal folds and thickness of muscularis: anterior segment, middle segment and posterior segment. Among these portions, the posterior intestinal segment was initially discovered as the preferential site of bacterial antigen uptake by Rombout *et al*. [11], where they classified the ‘second gut segment' in carp as playing a key role in antigen uptake and processing as well as the initiation of a systemic immune response [11]. All segments of the intestine comprise four concentric layers: serosa, muscularis mucosa, lamina propria and mucosa, which is similar to that of mammals [12]. The differences between fish and mammalian intestinal architecture include the unclear segmentation and the little difference in morphology, along with the fact that fish do not have characteristic intestinal mucosal crypts [10]. The serosa consists of a thin coating of squamous epithelium and connective tissue, which lies outermost of the intestine; next to the serosa is the muscularis, filled with muscle fibre sheets; the submucosa is a concentric layer that is made up of loose connective tissues, and is not present in all teleost species; the inner layer is mucosa, comprising an epithelial lining with distinct basement membrane and a lamina propria, which is a thin connective tissue that acts as a physical–chemical barrier [13–17]. Epithelial cells in mucosa defend against outbreaks and dietary antigens, which are essential for the intestinal mucosal immunity [14]. Moreover, goblet cells in the mucosa layer also play a critical role in protecting the intestine barriers through the production of mucus, as well as the secretion of antimicrobial proteins, chemokines and cytokines that stimulate the intestine local immunity and protect the barriers [18]. Several techniques have been used to study the intestinal health and barrier functions in fish, such as physiological measures obtained both *in vivo* and *in vitro*, histological techniques including transmission electron microscopy (TEM), the use of antibodies for immunohistochemistry, immunocytochemistry and western blots, as well as measurements of mRNA levels [10,14,19–22]. However, studies on the different compartments of the intestine and their specific functions in teleosts are still lacking.

Spatial transcriptomics (ST) can map transcriptional signatures to distinct geographical regions that are vital in development, where patterning and location-specific morphogen gradients shape organogenesis [23]. It combines high-resolution tissue imaging with unbiased spatially defined RNA sequencing (RNA-seq) using barcoded spots (100 µm) on glass slides [24]. Therefore, it is an attractive approach to resolve spatial differences in gene expression in different regions, yielding a substantially different, richer dataset than more traditional transcriptome analysis. The intestine offers a unique opportunity to investigate its functions because of its spatial organization [25]. A recent study characterized the transcriptomic landscape of human intestinal development, charting morphogenesis across time, locations and cellular compartments [26]. Parigi *et al*. [25] used ST to define how the transcriptomic landscape was spatially organized in the steady state and healing murine colon. They spatially placed cell populations and pathways that might play pivotal roles in driving tissue response to damage [25,27]. Additionally, ST has transformed our understanding of tissue functional organization and cell-to-cell interactions *in situ*. However, the exact molecular mechanism response process in fish, especially in the intestine, is not completely understood.

*Sebastes schlegelii*, known as black rockfish, is an important mariculture fish in East Asian countries due to its high economic and ecological values. With the increase of farming scale, various diseases caused great economic losses to the region's aquaculture [28]. Therefore, studies on the immunity of this species can expand our understanding of its immune response and regulatory mechanisms, and are also helpful to guide the prevention and control of diseases. Previous studies have reported that numerous immune-related genes (e.g. *IκB,* apoptosis-stimulating of p53 protein 1 and interleukin-1) and KEGG pathways (e.g. NF-κB signal pathway, pathogen recognition receptors related to signalling pathways and chemokine signalling pathway) were induced in the intestine once *S. schlegelii* was infected with bacteria [22]. However, these studies lacked the spatial resolution describing where genes were expressed. Emerging technologies for genome-wide ST offer significant potential for providing detailed molecular maps that overcome the limitations of high-throughput sequencing [24,29].

Therefore, we used the 10× Genomics Visium platform to generate spatial maps of gene expression in the posterior intestine of *S. schlegelii* across infection time, locations and cellular compartments to further expand our understanding of gene expression. Meanwhile, we also focused on the microbiota compositions among healthy and infected posterior intestine, and specifically discussed how ST has advanced knowledge of the immune mechanism that responds to *E. piscicida* infection in the posterior intestine of *S. schlegelii*, which also provides a theoretical basis for the spatial distribution of immune genes and changes in bacterial flora in other teleosts in the process of resisting pathogens.

## 2. Results

### 2.1. Transcriptome expression profiles and transmission electron microscopies of the posterior intestine of *Sebastes schlegelii*

**Figure 1.**
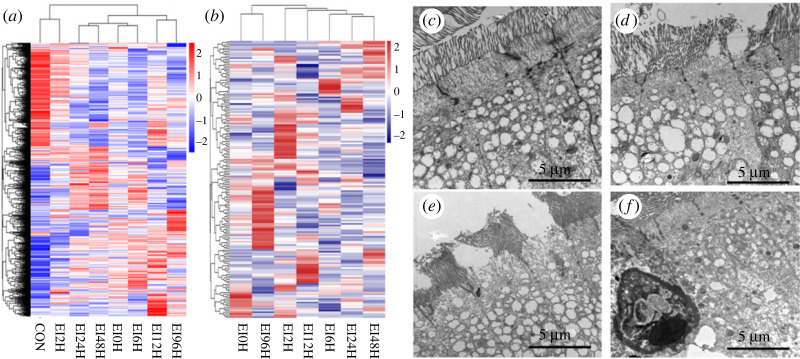
Transcriptome expression profiles and TEMs analysis of the posterior intestine in healthy and infected conditions. (*a,b*) Expression profiles of all detected genes and immune genes. (*c–f*) TEMs of normal intestine and the three infected intestinal tissues.

To explore the differences of gene expression profiles of *S. schlegelii* posterior intestine when it was exposed to *E. piscicida*, we applied unique molecular identifier to construct transcriptomes at different time points (0 h, 2 h, 6 h, 12 h, 24 h, 48 h and 96 h) (electronic supplementary material, table S1). All detected genes and immune-related genes in the heatmap showed that the changes of gene expression were obvious after infection at 6 h, 24 h and 96 h post-infection time points (figure 1*a,b*). Moreover, control (CON) and three infected posterior intestinal tissues (6 h -EI6H, 24 h -EI24H and 96 h -EI96H) were dissected for transmission electron microscopic analysis to observe the histopathological changes in the posterior intestine. Transmission electron microscopics (TEMs) analysis showed that different regions of layers arranged in a healthy condition (figure 1*c*). When infection time increased, the integrity of the intestinal structures was changed, the villi of the intestinal epithelial layer was broken and shed, and the vacuolization of cells became more serious at EI6H, EI24H and EI96H (figure 1*d–f*). Our results indicated that the destruction of *E. piscicida* to posterior intestine of *S. schlegelii* was in a time-dependent manner. Combining these two results, samples in control and infected groups at 6 h, 24 h and 96 h were finally selected to analyse the spatial expression of genes in the posterior intestine of *S. schlegelii*.

### 2.2. The distinct molecular regions of *Sebastes schlegelii* at healthy conditions

**Figure 2.**
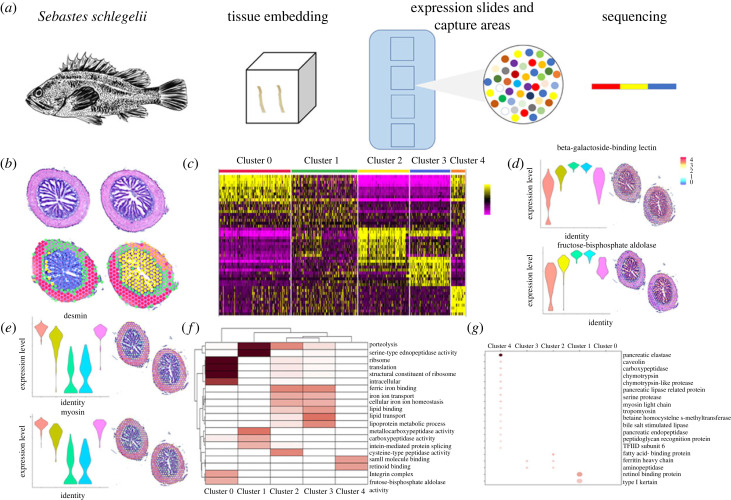
ST reveals molecular regionalization of the posterior intestine of *S. schlegelii* in healthy state. (*a*) Schematics of the experiment: intestine from the healthy *S. schlegelii* was processed as a Swiss roll for ST with 10× Genomics Visium platform technology (*n* = 2 Swiss roll). (*b*) Intestine Swiss rolls shown in haematoxylin and eosin (H&E) staining (up) and with each ST spot colour coded based Seurat. ST spots belonging uniquely to different clusters are coloured in red, green, yellow, blue and orange for Clusters 0, 1, 2, 3 and 4, respectively. (*c*) Heatmap of the expression of top genes among different clusters. (*d*) Spatial distribution of two genes in the mucosa layers. (*e*) Spatial distribution of 2 genes in the muscular and serosa layers. Each ST spot is assigned a colour-coded score based on the expression of the genes defining each cluster. (*f*) GO enrichment analysis using these genes in five clusters. (*g*) KEGG enrichment analysis using these genes in five clusters.

To characterize the transcriptomic signatures of the posterior intestine of *S. schlegelii* at healthy condition, we processed frozen posterior intestine for ST using the 10× Genomics Visium platform (figure 2*a*). Based on the intensity of the fluorescence signal and the degree of dispersion of the fluorescence signal, 18 min was selected as the optimal permeabilization time (electronic supplementary material, figure S1). After sequencing, 104.8 Gb raw data were generated from healthy posterior intestine tissue. Following the quality control, 82% of the filtered dataset could mapped onto the genome (electronic supplementary material, table S2, figures S2 and S3). The non-variable count was 5909 and variable count was 3000 (electronic supplementary material, figure S4). Among these variable genes, we found that these datasets contained 626 individual spots with an average of about 920 genes per spot. Overall, five basic structural transcriptomic landscapes were identified in the posterior intestine based on the distribution of spots. Among these, spots in Clusters 2 and 3 seemed to locate at the intestinal epithelial cells in mucosa layer, spots in Clusters 0 and 4 were distributed in both muscular and serosa layers, and spots in Cluster 1 were distributed in the middle of these two regions (figure 2*b*; electronic supplementary material, figure S5). Both correction analysis and expression patterns showed that the genes in Clusters 2 and 3 clustered together, and genes in Clusters 0 and 4 clustered together (figure 2*c*). To better visualize the molecular regionalization of each cluster, we analysed the top contributing genes for each cluster. Among these five clusters, 285 genes were significantly differentially expressed; 115, 2, 78, 64 and 26 significantly differentially expressed genes were found in each cluster. Among the genes driving Clusters 2 and 3, we found 143 genes that were highly expressed in the mucosa layer including CCL25, claudin-15a, athepsin B, cathepsins, etc. (figure 2*d*; electronic supplementary material, figure S6 and table S3). By contrast, genes related to structure maintenance such as caldesmon, caveolin, clusterin, myocilin, myosin, etc., were enriched in the muscular and serosa layers (Clusters 0 and 4) (figure 2*e*; electronic supplementary material, figure S7), and keratin 94 and retinol-binding protein 4 were enriched in the middle place (Cluster 1). ST analysis showed that there existed structural and functional differences in specific compartments of the posterior intestine of healthy condition.

Gene ontology (GO) enrichment analysis of genes in Clusters 2 and 3 is specialized in ion transport and lipid metabolism, while genes in the mucosa serosa layers are specialized in intein-mediated protein splicing, serine-type endopeptidase activity, carboxypeptidase activity, metallocarboxypeptidase activity and proteolysis, translation, structural constituent of ribosome, ribosome intracellular, integrin complex, fructose-bisphosphate aldolase activity, and genes in Cluster 1 took functions in small molecule binding and retinoid binding (figure 2*f*). KEGG enrichment analysis showed genes in the muscular and serosa layers were related to structure maintenance genes and enzymes (figure 2*g*), indicating functional differences among in mucosa, muscular and serosa layers.

### 2.3. Molecular landscape of posterior intestine following bacterial challenge

**Figure 3.**
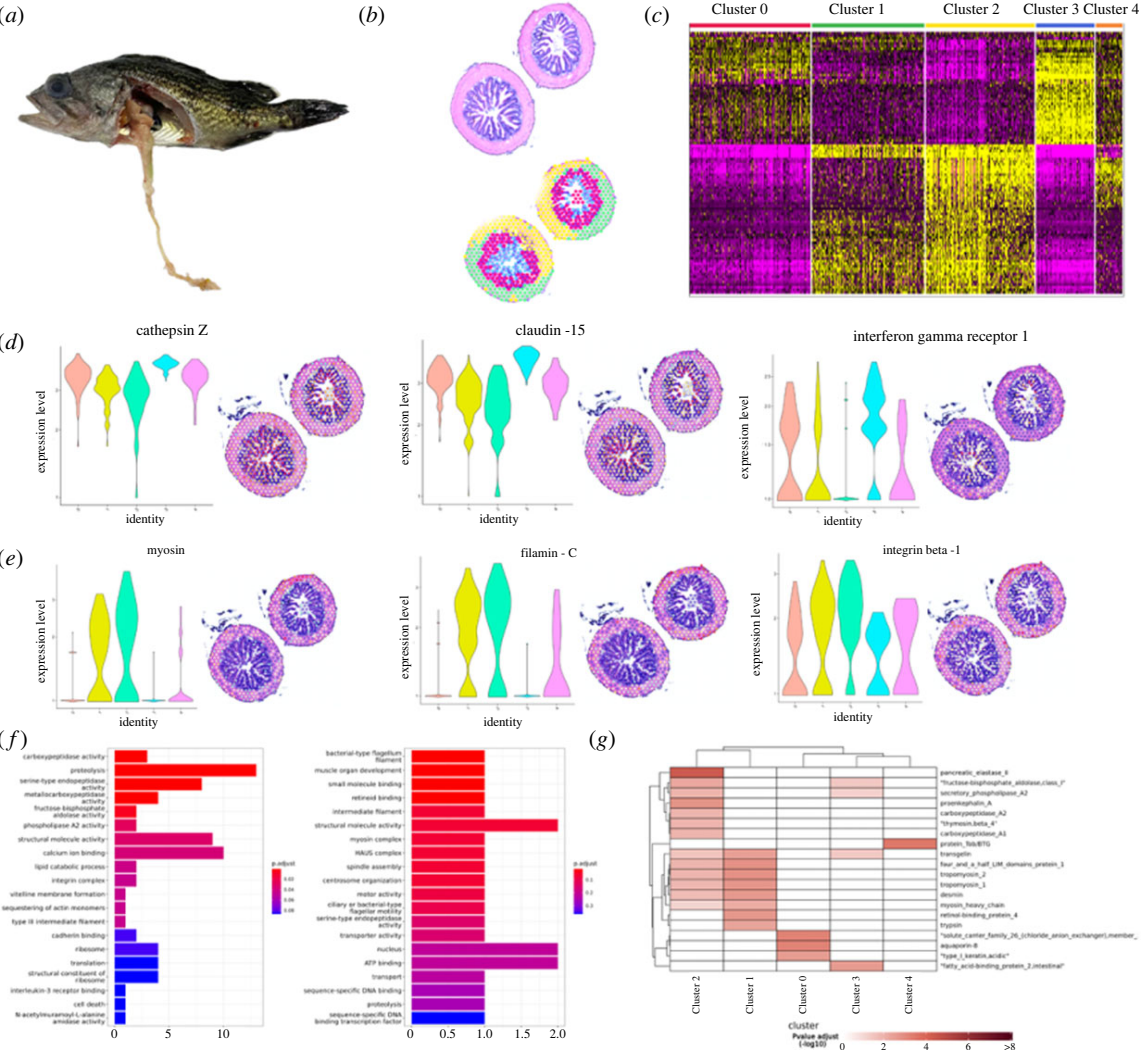
ST reveals molecular regionalization of the posterior intestine of *S. schlegelii* in EI6H. (*a*) Morphology of infected *S. schlegelii* in EI6H. (*b*) Intestine Swiss rolls shown in H&E staining (up) and with each ST spot colour coded based Seurat. ST spots belonging uniquely to different clusters are coloured in red, green, yellow, blue and orange for Clusters 0, 1, 2, 3 and 4, respectively. (*c*) Heatmap of the expression of top genes among different clusters. (*d*) Spatial distribution of three genes in the mucosa layers. (*e*) Spatial distribution of three genes in the muscular and serosa layers. Each ST spot is assigned a colour-coded score based on the expression of the genes defining each cluster. (*f*) GO enrichment analysis using these genes in five clusters. (*g*) KEGG enrichment analysis using these genes in five clusters.

For profiling the spatial transcriptomic landscape in posterior intestine of *S. schlegeii* following bacterial infection, the infected posterior intestine tissues at different time points were taken for ST. The EI6H ST dataset consisted of 690 individual spots, with an average of approximately 1562 genes per spot (figure 3*a*). Five basic structural transcriptomic landscapes were identified including mucosa layer (Cluster 0 and 3), a mixture between serosa and muscular layers (Cluster 1, 2 and 4) (figure 3*b*). To better visualize the molecular compartmentalization among these three layers, we analysed the significantly expressed genes for each cluster of the muscular and mucosa layers, and the mixed signature between serosa and muscular layers (electronic supplementary material, figure S8). Overall, 249 genes were significantly differentially expressed. Among the genes driving Clusters 0 and 3, we found 136 genes located in the mucosa layers (figure 3*c*; electronic supplementary material, table S5 and figure S9). In Clusters 1, 2 and 4, respectively 9, 102 and 2 genes showed significant expression. We found that some immune-related genes such as myocilin, filamin-C and integrin beta-1 were highly expressed in the serosa and muscular layers, and genes such as cathepsin Z, claudin-15 and interferon-γ-receptor (IFN*γ*R1) were highly expressed in the mucosa layers (figure 3*d*; electronic supplementary material, table S6 and figure S10). GO enrichment analysis using the top contributing genes of the mucosa layers suggested that genes in this section of posterior intestine are specialized in proteolysis, structural molecule activity and serine-tyoe endopeptidase, etc., while genes in the mucosa and serosa layers are specialized structural molecule activity, nucleus and ATP binding (figure 3*e*; electronic supplementary material, figure S11). Meanwhile, KEGG enrichment analysis demonstrated that genes related to desmin, transgelin, carboxypeptidase and myosin heavy chain were mainly distributed in Clusters 1, 2 and 4. In addition, solute carrier family, aquaporin 8 and type I keratin were found in Clusters 0 and 3 (figure 3*f*; electronic supplementary material, figure S12).

**Figure 4.**
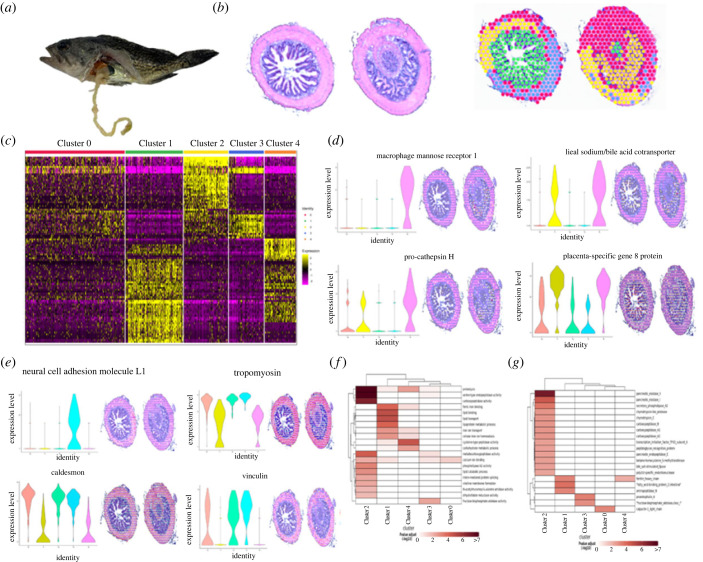
ST reveals molecular regionalization of the posterior intestine of *S. schlegelii* in EI24H. (*a*) Morphology of infected *S. schlegelii* in EI24H. (*b*) Intestine Swiss rolls shown in H&E staining (up) and with each ST spot colour coded based Seurat. ST spots belonging uniquely to different clusters are coloured in red, green, yellow, blue and orange for clusters 0, 1, 2, 3 and 4, respectively. (*c*) Heatmap of the expression of top genes among different clusters. (*d*) Spatial distribution of four genes in the mucosa layers. (*e*) Spatial distribution of four genes in the muscular and serosa layers. Each ST spot is assigned a colour-coded score based on the expression of the genes defining each cluster. (*f*) GO enrichment analysis using these genes in five clusters. (*g*) KEGG enrichment analysis using these genes in five clusters.

For the E24H group (figure 4*a*), we found 720 individual spots mapping to the slice of the posterior intestine, with an average of approximately 582 genes per spot (E24H) (figure 4*b*). Differentially regulated genes per cluster were presented in a heatmap (figure 4*c*). Each cluster defined a different region of the posterior intestine. For instance, genes in Clusters 1 and 4 scattered expressions in the mucosa layer, whereas Clusters 2 and 4 mapped spatially to the muscular and serosa layers (figure 4*b*). Interestingly, genes defining Cluster 0 mixed during the infection process, which were found in all the sections of the posterior intestine. Both correction analysis and expression patterns showed that the Clusters 2 and 3 were clustered together. In contrast, the expression patterns of genes in Clusters 1 and 4 representing the mucosa layers did not cluster together. Overall, 152 genes showed significant expression at this time point. Among the genes driving Clusters 2 and 3, we found that most of the gene contents were similar to those in EI6H group. Additionally, macrophage mannose receptor 1 and pro-cathepsin H were found in the mucosa layer (figure 4*d*). In addition to genes related to muscle composition and cellular structure, we also identified tenascin, vinculin-like, CHL1 and regulator of G-protein signalling 5-like driving Clusters 0, 3 and 4 (figure 4*e*). GO enrichment analysis showed that the detected genes were closely related to ion transport, glucose and lipid metabolism (figure 4*f*; electronic supplementary material, figure S13). KEGG enrichment of genes in Clusters 1 and 4 is specialized in fatty acid-binding protein 2, aminopeptidase and ferritin, and genes in Cluster 0 are specialized in calpactin (figure 4*g*; electronic supplementary material, figure S14).

**Figure 5.**
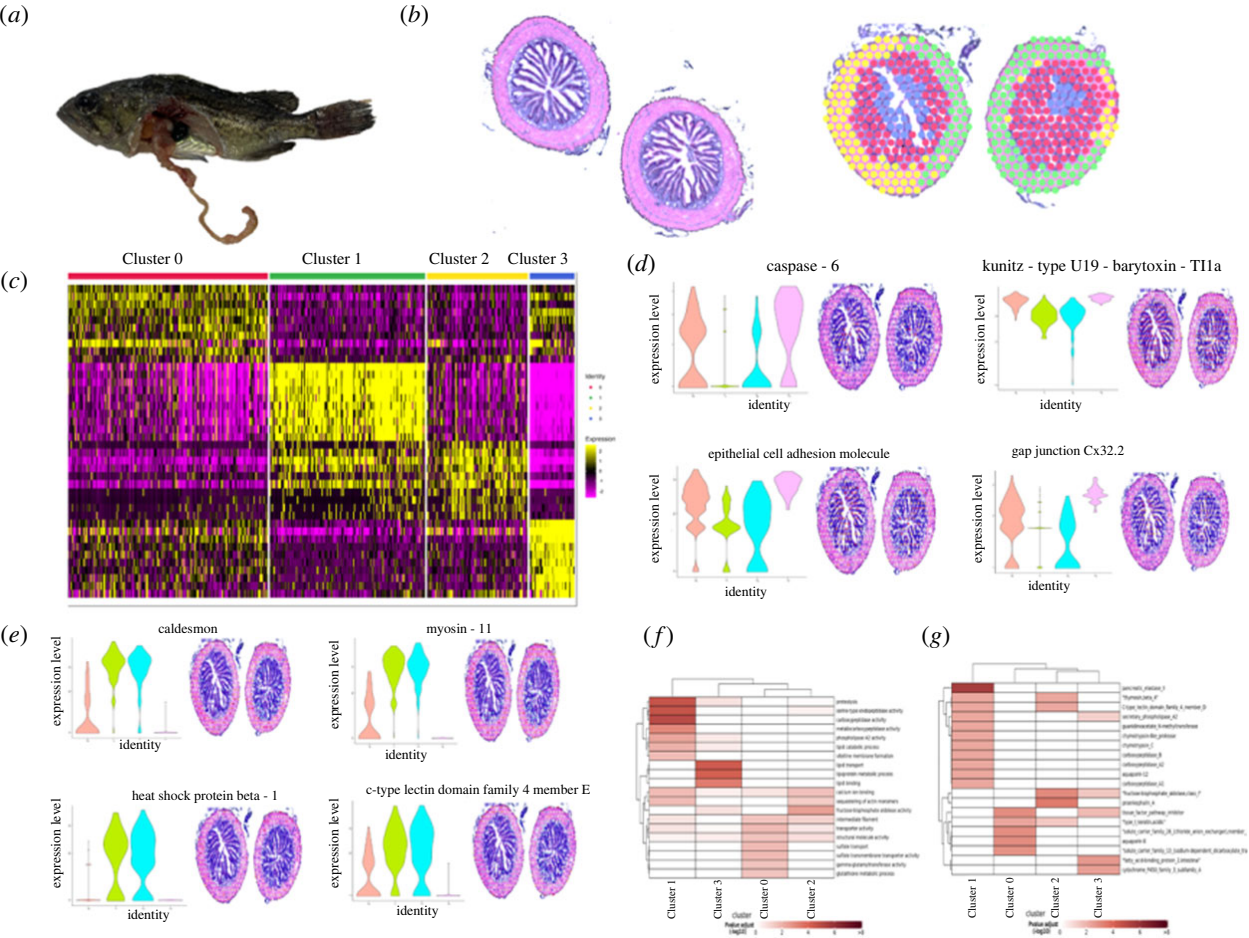
ST reveals molecular regionalization of the posterior intestine of *S. schlegelii* in EI96H. (*a*) Morphology of infected *S. schlegelii* in EI96H. (*b*) Intestine Swiss rolls shown in H&E staining (up) and with each ST spot colour coded based Seurat. ST spots belonging uniquely to different clusters are coloured in red, green, yellow, blue and orange for Clusters 0, 1, 2, 3 and 4, respectively. (*c*) Heatmap of the expression of top genes among different clusters. (*d*) Spatial distribution of four genes in the mucosa layers. (*e*) Spatial distribution of four genes in the muscular and serosa layers. Each ST spot is assigned a colour-coded score based on the expression of the genes defining each cluster. (*f*) GO enrichment analysis using these genes in five clusters. (*g*) KEGG enrichment analysis using these genes in five clusters.

For the EI96H-infected samples (figure 5*a*), the resulting dataset consists of 523 individual spots, with an average of approximately 1037 genes per spot (electronic supplementary material, figure S15). Different with the previous samples, four clusters were found in the posterior intestine (figure 5*b,c*). Among the genes driving Clusters 0 and 3, we found several immune-related genes such as claudin-15a, connexin, mucin-2, *CCL25*, epithelial cell adhesion molecule (EpCAM), cathepsin Z, ladinin-1, occludin, IFN*γ*R1, claudin-34, *CXCL5* and caspase-6 exhibited high expression levels (figure 5*d*; electronic supplementary material, table S9 and figure S16). Among the genes driving Clusters 1 and 2, we found caldesmon 1b, C-type lectin, HSPB1, myosin, regulator of G-protein signalling 5-like, transgelin and tropomyosin beta chain (TPM2) were highly expressed (figure 5*e*; electronic supplementary material, table S10 and figure S17). The spatial mapping results showed that genes in Clusters 0 and 3 mapped in the mucosa layer and genes in Clusters 1 and 2 mapped in the muscular and serosa layers. We next examined the GO terms and KEGG pathways enriched in each cluster. Functional enrichment analysis using genes of the muscular and serosa layers suggested that genes were specialized in several processes such as lipid metabolic process and glutathione metabolic process (figure 5*f*), while genes in the mucosa layer were specialized in proteolysis, phospholipase A2 activity, lipid catabolic process, muscle organ development and capsule polysaccharide biosynthetic process (figure 5*g*).

### 2.4. Uniform manifold approximation and projection analysis revealed transcriptomic regionalization during bacterial infection

**Figure 6.**
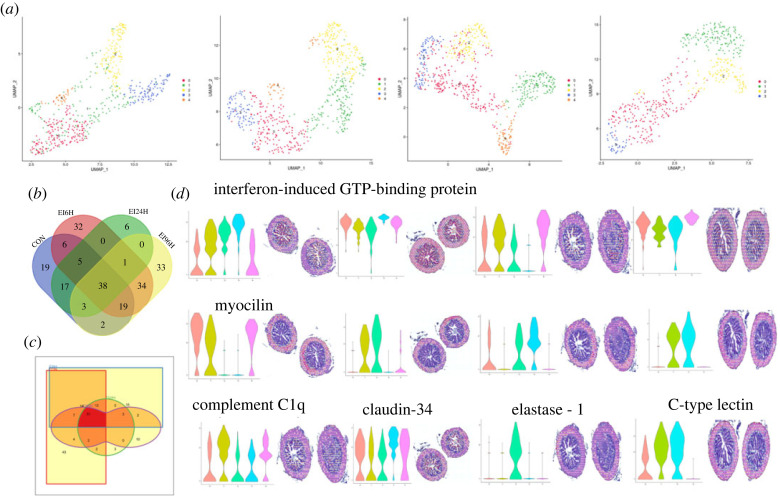
Non-negative matrix factorization reveals distinct molecular patterns during the infection process of infection. (*a*) UMAP representation of clusters in control and three infected groups. (*b*) Venn diagram of common and specific genes in the mucosa layers. (*c*) Venn diagram of common and specific genes in the muscular and serosa layers. (*d*) Expression patterns of common and specific genes in the mucosa, muscular and serosa layers. Horizontal coordinates represent expression level and vertical coordinates represent identity.

According to the results of uniform manifold approximation and projection (UMAP), the spots in CON, EI6H and EI24H group were grouped into five clusters, and the spots in EI96H were grouped into four clusters (figure 6*a*). In detail, UMAP results of CON showed that each cluster clustered respectively in CON, except for Cluster 1, which were scattered in other clusters. Similar situations were found in Cluster 4 of EI6H, Cluster 3 of EI24H and Cluster 2 of EI96H. Owing to their important roles in response to pathogenic bacterial infections and structural maintenance, we focused on these clusters in different sections in the posterior intestine. In mucosa layer, Clusters 2 and 3 were characterized by genes involved in fatty acid metabolism in CON. Pathway analysis revealed these two clusters were associated with processes involving in ion transport and lipid metabolism. Similarly, genes in Clusters 0 and 3 in EI6H and EI96H, genes in Clusters 1 and 4 in EI24H were located in the mucosa layer. In the mucosa layers of all the four samples, we found that 38 genes were shared in these four samples. These genes were associated with glucose metabolism (e.g. fructose-bisphosphate aldolase B), lipid metabolism (fatty acid-binding protein, intestinal), ion transport (e.g. SLC13A2, NHE-RF3), components of epithelial cells (keratin, cadherin-related family member 2) and immune genes (e.g. cathepsin Z) in epithelial cells. Here 32, 6 and 33 genes were only found in EI6H, EI24H and EI96H, respectively (figure 6*b*; electronic supplementary material, figures S11–S14). The majority of genes in the EI6H treated group were annotated as lysophospholipase D, NPC1L1, sialidase-1 and SIDT2, as well as genes involved in immunity or maintenance of cellular structure such as cathepsin D and claudin-34. Apoptosis-related genes such as MARVEL domain-containing protein 3. The majority of genes in the EI24H-treated group were annotated as pro-cathepsin H, histone H3.3, macrophage mannose receptor 1, globoside alpha-1, 3-*N*-acetylgalactosaminyltransferase 1-like, EDF1, insulin 5a. The majority of genes in the EI96H-treated group was immune-related, including caspase-6, claudin-34, *CXCL5*, EpCAM, IL-13R*α*1, ladinin-1 and CD46, as well as LC3B and cytochrome P450. In addition, we found CMP-*N*-acetylneuraminate-beta-galactosamide-alpha-2,3-sialyltransferase 1-like was only in three infected groups. By contrast, Clusters 0, 1 and 4 of CON, clusters 1, 2 and 4 of EI6H, clusters 0, 2 and 3 of EI24H, clusters 1 and 2 of EI96h were also drastically enriched in the muscular and serosa layers. Overall, 51 common genes in these clusters distributed in muscular and serosa layers were characterized by genes involved in calcium ions and metabolism (e.g. calcium-binding protein P, caldesmon 1b and calmodulin) and bacterial recognition (e.g. PGRPs, beta-galactoside-binding lectin), the formation of muscular layers and movement (e.g. myocilin, secretagogin). Especially, clusters in the muscular and serosa layers were characterized by the expression of genes involved in immune response (e.g. complement C1q subcomponent subunit A (C1qa) and C-type lectin), which indicated a response to the invasion of bacteria (figure 6*d*; electronic supplementary material, figures S15–S18).

### 2.5. Spatial transcriptomics of immune-related genes

**Figure 7.**
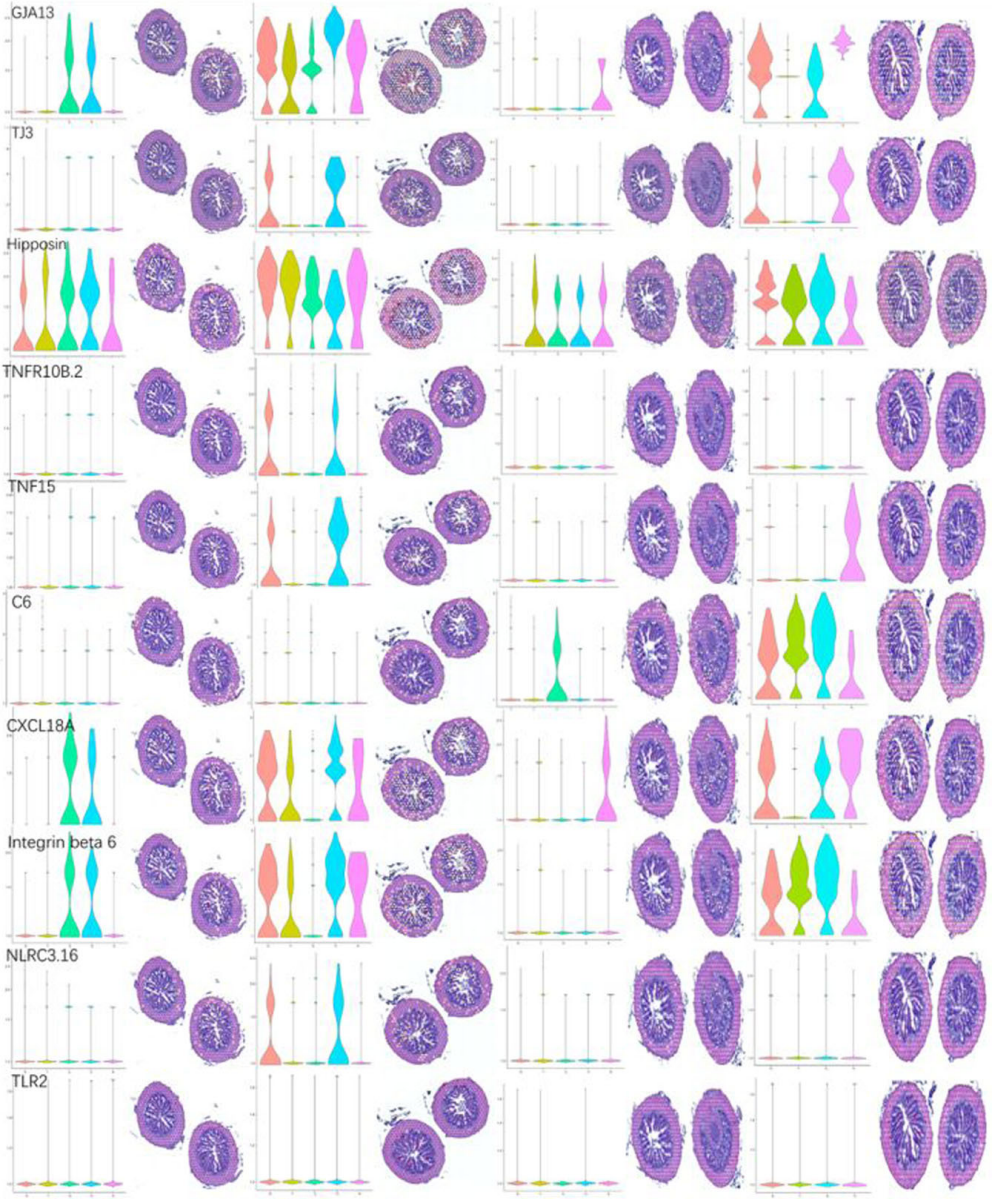
ST reveals molecular regionalization of the immune-related genes in the posterior intestine of *S. schlegelii.* ST landscape of immune-related genes in the intestine of *S. schlegelii.* Horizontal coordinates represent expression level and vertical coordinates represent identity.

Intestine serves as a site of immune cells priming at birth, which can prevent pathogen adhesion and invasion. However, the ST of these immune-related genes and their responding mechanisms in *S. schlegelii* has not been completely elucidated. Therefore, we explored the spatial distributions and expression patterns of these immune-related genes in the posterior intestine of *S. schlegelii* (figure 7; electronic supplementary material, figure S22)*.* The results showed that PRRs such as TLR2 and NLRC3.16 were highly expressed at the early infection stage, which mainly distributed in the mucosa layers. In the complement system, *C1qa, C1qb, C1qc* and *CLU* were highly expressed in all the infected stages. By contrast, *C1qbp, C1q12.2, C1q12, CFD* and COLLECTIN were highly expressed in the relatively late stage of infections such as EI24H and EI96H. Most of the genes in the complement system were scattered in the muscular and serosa layers. Meanwhile, we found that integrins distributed in the mucosal layers such as integrins alpha 3 and beta 6 at EI6H group. Similar situation was found in *TNF15* and *CCL25*. In addition, antimicrobial peptides such as hipposin and alpha defensin were distributed in mucosa, muscular and serosa layers, and were highly expressed in the early stages of infections. Genes involved in cell-to-cell connections were also induced during infection. Most of genes related to gap junctions were distributed throughout the posterior intestine, while genes related to tight junctions were scattered in the mucosa layers. The expressions and distributions of these immune-related genes demonstrated that they all participated in the infection of bacterial. Taken together, genes in complement system, chemokines and antimicrobial peptides were distributed in all three layers of the posterior intestine, while TLR, NLR, integrin, tumour necrosis factor mainly distributed in the mucosa layers.

### 2.6. Spatial transcriptomics of genes in immune-related KEGG pathways during bacterial infection

**Figure 8.**
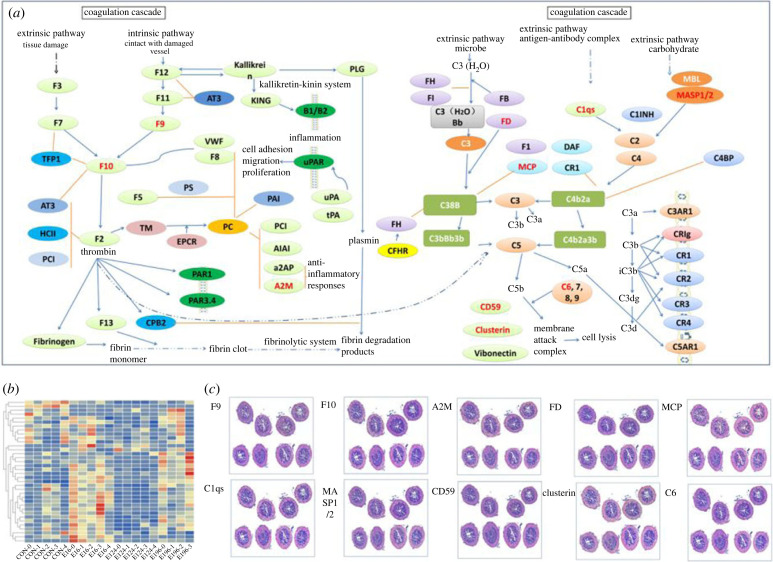
KEGG pathway activities during bacterial infection in the intestine of *S. schlegelii.* (*a*) Complement and coagulation cascades pathways. (*b*) Expression patterns of genes in complement and coagulation cascades pathways; (*c*) ST landscape of genes (marked with red) involved in the complement and coagulation cascades in the intestine of *S. schlegelii.*

To explore the spatial distribution of genes involved in immune-related signalling pathways in the posterior intestine, related signalling pathways were selected to study the distribution of these genes. First, we compared the expression patterns and distributions of genes in these KEGG pathways by Seurat (figure 8; electronic supplementary material, figure S23). We found genes in these immune-related KEGG pathways showed similar expression patterns at 6 h and 96 h following infections. We observed that pathways including NLR signalling pathway, RIG signalling pathway, C-type lectin receptor signalling pathway, TLR signalling pathway were associated with clusters comprising damaged epithelium, muscular and serosa layers (e.g. Clusters 0, 1, 2, 3 and 4 at EI6H). The spatial expression of genes in pathways related to innate immunity such as B-cell receptor signalling pathway, T-cell receptor signalling pathway, Fc epsilon RI signalling pathway, Fc gamma R-mediated phagocytosis, antigen processing and presentation were also investigated (electronic supplementary material, figure S23). For example, genes in B-cell receptor signalling pathway such as MALT1 PE were associated with the damaged epithelium (e.g. Clusters 0 and 3 at EI6H and EI96H). In addition, Cytosolic DNA-sensing and *IL-17* signalling pathways were associated with innate immunity and inflammation, respectively. It is worth noting that, Cytosolic DNA-sensing and *IL-17* signalling pathways were homogeneously active in control, whereas during infection, their activation was higher within the mucosa layers. We also noticed that plenty of complement and coagulation cascades including *F9, F10, A2M, C1qs, C6* and *CD59* were highly expressed in all the infected stages, and genes in the complement and coagulation cascades were distributed in all the layers of posterior intestine (figure 8).

### 2.7. Comparative analysis of 16S rRNA genes in the posterior intestine of *Sebastes schlegelii*

**Figure 9.**
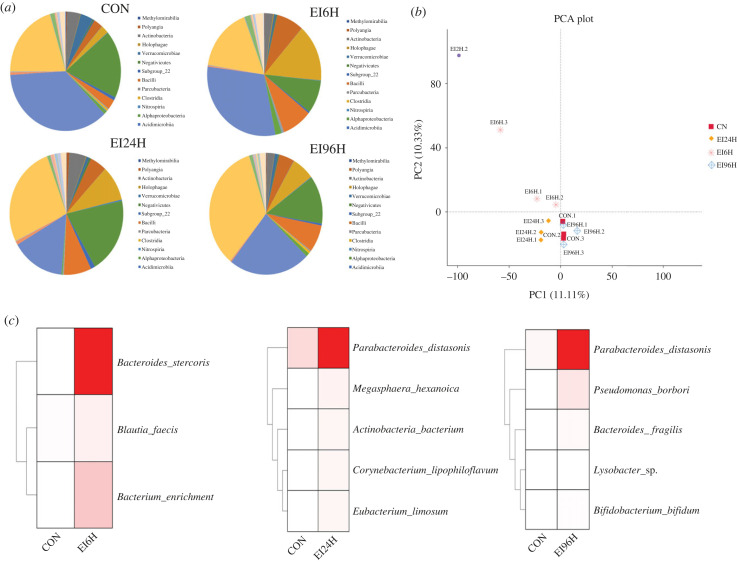
Comparative analysis of 16S rRNA genes in the posterior intestine of *S. schlegelii.* (*a*) The bacteria community among CON, EI6H, EI24H and EI96H. (*b*) The PCA of OTUs of three replicates from each group. (*c*) Several representatives of bacteria in the infected groups.

To explore the changes of intestinal bacterial community diversity in the infected posterior intestine, we amplified the V4-V5 region of 16S rRNA. The sequencing results showed that the species rarefaction curve tends to be flat, indicating that the depth of sequencing met the requirement for subsequent analysis. First, we detected and compared the taxa abundance in the intestinal bacterial community diversity between CON and the infected groups based on their community abundance and diversity index (figure 9*a*). *E. piscicida* was not detected in the control group. With the increase of infection time, the abundance of *E. piscicida* increased firstly and then decreased*. E. piscicida* in EI96H decreased to 40th and 38th of those in EI6H and EI24H, respectively. We also found that samples had lower alphadiversity in CON group than samples in the infected groups at the genus levels based on the index of Chao 1, Observed_otus, Pielou_e, Shannon and Simpson. For instance, the mean of Chao 1 increased as the infection time, with the largest differences observed at 6 h time point (*p*-value < 0.001). Shannon indices varied from 5.96 to 7.72. The CON had the lowest Shannon index and Chao1 richness, with 5.96 and 851.41 OTUs, respectively (electronic supplementary material, table S19). Moreover, NMDS analysis was used to analyse the β-diversity among different groups. The PCA of OTUs indicated that three replicates from the same conditions grouped together (figure 9*b*). In detail, bacterial communities from EI96H were close to that in CON, while distinct separation was found between those from EI6H and EI24H, demonstrating that the composition bacterial communities were affected by infection. In the CON group, Cyanobacteriia, Gammaproteobacteria and Alphaproteobacteria phyla predominated, as was also the case in EI6H and EI96H groups. In addition, we noticed that species in Clostridia increased in the infected samples. Among these bacteria, we found that *Bacteroides stercoris* (*p* < 0.05)*, Bacterium enrichment* (*p* < 0.05)*, Blautia faecis* (*p* < 0.05) increased in the infected groups (EI6H). Moreover, we found abundance increased in species such as *Parabacteroides distasonis* (*p* < 0.01), *Megasphaera hexanoica* (*p* < 0.05), *Corynebacterium lipophiloflavum* (*p* < 0.05), *Eubacterium limosum* (*p* < 0.05), *Actinobacteria bacterium* (*p* < 0.05) in EI24H group. Comparatively, the 96 h infected group comprised a number of major species, such as *Bifidobacterium bifidum* (*p* < 0.05), *Bacteroides fragilis* (*p* < 0.05), *P. distasonis* (*p* < 0.01), *Lysobacter* sp. (*p* < 0.05), *Pseudomonas borbori* (*p* < 0.05) (figure 9*c*).

## 3. Discussion

The intestine contains the largest number of immune cells in the organisms, and is continually exposed to a wide range of antigens and potential immune stimuli. There is an increasing awareness of how the contents of the intestine, such as the commensal bacteria and dietary constituents, influence physiological and pathological processes throughout the body [4]. Such vital roles have led to a surge in the number of studies investigating the immune response of the intestine. Recent technological advances in combination of the sequencing and image can make genes spatially mapped onto the histological brightfield images in different organisms, as well as in the progress of development and a range of disease contexts [30,31]. For example, a recent study characterized the intestinal morphogenesis through time, and identified 101 cell states by linking single-cell RNA sequencing and ST [26]. Furthermore, Parigi *et al*. used ST to define the genes and their distribution in the healing process of murine colon, and found that the decreased *p53* can increase the presence of proliferating epithelial stem cells [25]. These studies offered valuable resources for investigating the gene regulation networks and spatial transcriptomic landscape of the human and mouse intestine, and provided references for the study of the intestine of other non-model species.

Teleosts live in microbial-rich aquatic environments, where damage to the intestinal barrier leads to trigger a systemic pathological response, which will lead to a major impact on fish health. Therefore, previous studies have characterized the transcriptomes of intestine response to pathogens in teleost [19,22]. Previously, we found that the integrity of the intestinal mucosa structure changed, including hyperplasia of the mucosa, thickening of the lamina propria, epithelial cell shedding, mucosal fold breakage, increase in goblet cells, and changes in autolysis and necrosis after infection with *E. piscicida,* thus we constructed the temporal transcriptomic dynamics of mRNAs, circRNAs and miRNAs to explore its response mechanisms in the intestine of *S. schlegelii* [22]. Although we found a large number of immune-related genes were significantly changed during infection, the spatial distribution of gene expression locations cannot be studied by regular RNA-seq analysis. Therefore, we performed spatial transcriptomic analysis in *S. schlegelii* following *E. piscicida* infection to confirm the spatial localization of gene signatures and the distribution of immune-related genes and KEGG pathways in response to pathogens.

In this study, we overcome the limitations of normal transcriptomes and uncover a view of the molecular regionalization of the posterior intestine of *S. schlegelii* using ST. In the healthy condition, we identified a previously unappreciated molecular regionalization of the posterior intestine. Overall, five basic structural transcriptomic landscapes were identified in mucosa layer, in a mix of muscular and serosa layers, and in the middle of the two regions. The intestinal mucosal barrier plays a crucial role in maintaining host health by preventing the invasion of foreign substances. In the mucosa layer, we identified 143 genes that were highly expressed including several immune-related genes, which is in line with the immune function of the intestinal mucosal layer published previously. These immune-related genes such as β*-*chains of MHC class II, FcγBP, ferritin, tight junction, type I keratin and plastin indicated that they existed in this section of posterior intestine and prepared for the immune process when the host was infected by pathogens or protect epithelial tissue cells. In addition to the identification of immune-related genes in mucosa, we also found some genes that may relate to the epithelial cell proliferation, differentiation and migration, such as EPS8, which was distributed in the mucosa layer, and existed as a dimer and binds to the juxtamembrane region of the EGFR [32]. We speculated that it played an important role in epithelial cell proliferation and migration. Additionally, PLAC8 has been reported to be involved in early embryonic development in zebrafish and placental development in mice. Our results demonstrated that it is also distributed in the mucosal layer of the fish intestine. Therefore, we hypothesized that it is related to the proliferation and development of intestinal epithelial cells in fish.

In addition to the mucosa layer, the muscular and serosa layers separate the intestine from the surrounding peritoneal cavity. We observed different types of genes with structures and functions in muscular and serosa layers. Most genes related to muscle composition and structural maintenance, such as alpha-actinin-1, collagen alpha-2 chain and desmin, act as strong filament nucleators in muscular cells [33]. We also found genes with signal transduction and cell adhesion. For example, Junctophilin has been reported to be critical in coordinating the maturation and maintenance of cellular ultrastructure, ensuring calcium signalling and normal excitation–contraction coupling. Usually, junctophilin 2 was found in cardiac muscle [34]. In our study, junctophilin 2 was found in the posterior intestine muscle of fish. In addition, we found genes in the muscular and serosa layers that may be linked with immune function. Based on this, we speculated that genes in the muscular layer also may play immune roles in fish. We found NCAM located in the muscular and serosa layers in our study, which was previously found on early embryonic cells that playing an important role in cell morphogenesis, adhesion between neurons, and adhesion between neurons and muscles [35]. Therefore, we speculate that NCAM gene plays an important role in the morphogenesis and adhesion of intestinal muscle cells in teleost. ST analysis showed that there existed structural and functional differences in specific compartments of the posterior intestine in healthy condition. The function of the intestinal immune system in a healthy state is to maintain physiological functions under the constant bombardment of environmental substances, which is consistent with a previous study [25]. Spatial transcriptome analysis not only helps us identify which genes respond to pathogen invasion in the mucosa layer, but also helps us identify where in the posterior intestine these genes are functioning.

The intestine relies on the multi-layered structures and constant regeneration of the intestinal epithelium to maintain its function and homeostasis. Breakdown in its structures may lead to pathogen translocation and the development of chronic intestinal pathologies, such as inflammation [25]. Therefore, the intestinal barrier must rapidly adapt to establish corresponding response mechanisms to maintain intestinal homeostasis and balance. By comparing ST of the posterior intestine of *S. schegelii* under healthy conditions and upon bacterial infection, we identified and spatially mapped transcriptional signatures of tissue response processes and immune cell activation/recruitment. Overall, we identified that 38 common genes were shared in these four samples in the mucosa layers. These genes were associated with glucose metabolism, lipid metabolism, ion transport, components of epithelial cell and immune genes in epithelial cells. In addition, 32, 6 and 33 genes were uniquely found in EI6H, EI24H and EI96H, respectively. The majority of genes in the infected groups were immune-related. Among these genes, mannose receptor can recognize and bind specific carbohydrate molecules through the extracellular domain, and plays a role in recognizing pathogens, presenting antigens and maintaining homeostasis [36]. Macrophage mannose receptor 1 in the mucosa layers in fish also played a role in preventing bacterial invasion when bacteria invaded or colonized the intestine. Moreover, cathepsin D distributed in almost all cells and tissues of mammals with the function to mediate apoptosis [37]. The induced cathepsin D in the infected samples in the mucosa layers suggested that the apoptosis process was activated once the intestine was invaded. It also has been demonstrated that the balanced expression of cytokines that mediate inflammation and repair was critical in epithelial barrier function. Our study showed that the regulation of *IL-13* receptor subunits in the mucosa layer of the posterior intestine was closely related to the inflammatory process. In our study, we found that caspase-6 was mainly distributed in the mucosa layers and was induced by bacterial infection. We hypothesized that capase-6 may contribute to the defence of pathogens through promoting the activation of programmed cell death pathways and played an essential role in host defence against pathogen infection, as previously reported [38]. In addition to these immune-related genes, we noticed that genes related to cell differentiation and connectivity also existed in mucosa layers. For example, claudin-34 related to the process of tight junction was found in the mucosa layer of *S. schelegii* posterior intestine. Meanwhile, 51 common genes positioned in muscular and serosa layers were characterized by genes involved in calcium ion metabolism, bacterial recognition, formation of muscular layers and movement. Although initially muscular and serosa layers were considered as merely structural support, they were also actively involved in several immune responses. These findings allowed the identification of the mucosa layer, muscular and serosa layers depending on their localizations.

ST is a good choice for identifying region-specific marker genes for immune responses in the intestine. The available genes and signal pathways associated with immunity identified from RNA-seq and gene families allowed us to infer their involvement in the pathogen response process based on their localization in areas with distinct histological properties. We thus explored the spatial distribution and expression patterns of immune-related genes in the posterior intestine of *S. schlegelii.* The initial sensing of infection of cells in an organism is mediated by PRRs [39]. Our results showed that PRRs such as *TLR2* and *NLRC3.16* genes were highly expressed in the early stage, and mainly distributed in the mucosa layers, which can help the host quickly recognize the pathogens. Correspondingly, genes in NLR signalling pathway, RIG signalling pathway, C-type lectin receptor signalling pathway and TLR signalling pathway were scattered in mucosa, muscular layers and serosa layers, preparing for responding to infections at early stages of infection. Activation of the complement system can induce phagocytic clearance of pathogens, as well as cell lysis, which plays an important role in host defence and inflammation [40]. Plenty of complement genes and signal pathways were highly expressed in all the infected stages. Most of the genes in the complement system scattered in the muscular and serosa layers, while genes in the complement and coagulation cascades distributed in all the layers of posterior intestine. The genes in the muscular and serosa layers start to participate in the immune response. Moreover, antimicrobial peptides such as hipposin and alpha defensin were distributed in mucosa, muscular and serosa layers. In addition, we found that genes involved in cell-to-cell connection were also induced during infection, and distributed throughout the posterior intestine. In addition to immune genes, we also analysed the expression changes and distribution of genes in immune-related KEGG signalling pathways to estimate these signalling pathway activities. Among these pathways, B-cell and T-cell signal pathways were induced once infected by bacteria. Genes involved in B-cell and T-cell signal pathways were distributed in all the sections of intestine, and thus induced signalling cascades leading to the activation of a series of transcription factor families. In inflammatory factor-related signalling pathways, we noticed that genes in the IL-17 signalling pathway, such as *TNFAIP3, IKBa, CCL20, IL17RC, Rela, CXCL8*, were distributed in the mucosa layers, with high expression levels, especially at EI6H. It has been reported that IL-17-mediated inflammation is crucial for microbial clearance, while unrestrained IL-17 signalling can promote immunopathology [41]. Thus, we speculated that inflammation-related genes are overexpressed in the mucosal layer. Previous studies showed that the molecules in Fc epsilon RI signalling pathway can cause effects on various processes such as cytokine production and cell migration, growth and survival. Moreover, the Fc*γ*Rs can induce the secretion of *IL-4, IL-5, IL-6*, *IL-10*, *IL-13*, *INFγ*, *TNFα* and GCSF [42]. We found that these genes in Fc epsilon RI signalling pathways were induced in all the sections of the posterior intestine.

In addition to the vital roles of the innate immune genes, the intestine microbiota also assists the host to perform corresponding immune functions. Previous research has shown that the intestine harbours a diverse community of trillions of microbes, which have coevolved with the host immune system. Many of these microorganisms have vital functions for host physiology, but the host must remain vigilant to control the microbial community in order to maintain the symbiotic nature of the relationship [43]. The epithelial barrier integrity and the mucosal immune system were maintained by the intestinal microbiota via balancing host defence and microbial metabolites, components and attachment to host cells. Thus, understanding the interactions between intestine microbiota and immune cells in the intestine is critical for maintaining its homeostasis [44]. Therefore, we investigated the influence of *E. piscicida* on the posterior intestines of *S. schlegelii*, and analysed the changes in the microbiota composition in the posterior intestine of *S. schlegelii* at the species level following infection. Meta-analysis showed that the abundance of species did significantly differ between the control and the infected groups. In the posterior intestine of *S. schlegelii*, the number of *E. piscicida* increased significantly at the initial stage of infection (EI6H and E24H), and almost returned to the normal level at the 4th day of infection (EI96h). We also found that with the increase of infection time, the types of probiotics changed significantly. In our study, *B. stercoris* showed higher relative abundance in the infected samples at 6 h. As reported, CD8+ T-cell clones displaying the *B. stercoris* mimotope can cross-recognize a bacterial mimotope, and related with intestinal permeability and inflammation [45–47]. At the initial invasion stage, *E. piscicida* entered the posterior intestine of *S. schlegelii* and may change the intestine permeability, while increased *B. stercoris* can lead to immune response activation and contribute to the response to bacterial infection. In addition, the protective effects of *E. limosum*, together with its products SCFAs in gut inflammation, have been demonstrated *in vitro* and in murine models [47,48]. Therefore, it is speculated that with the increase of infection time, the *E. piscicida* that are not recognized and killed would further attack the posterior intestine, and intestinal immune cells can resist further infection of pathogenic bacteria by inducing corresponding inflammatory response by themselves or intestinal beneficial bacteria. Moreover, the infected 96 h samples showed a significantly higher relative abundance including *B. bifidum*, *B. fragilis* and *P. distasonis*. Usually, Bifidobacterium represents the most abundant bacteria in the human gut microbiota in healthy breast-fed infants. Many Bifidobacterium species such as *B. bifidum* have been described to produce beneficial effects, including antibacterial properties against pathogens [49,50], decreasing apoptosis of intestinal mucosal cells in premature infants with necrotizing enterocolitis [51], regulation of the host immune system [52] and reduction of inflammatory activity associated with chronic intestinal dysfunction [53,54]. Importantly, it has been demonstrated that *B. bifidum* can strongly inhibit the adhesion of enteropathogens [55]. Thus, we hypothesized that *B. bifidum* in the infected posterior intestine of *S. schlegelii* may participate in the inhibition of adhesion of pathogens. Among the changing flora, we found the abundance of *B. fragilis* was increased in infected sample at 96 h, which can produce unique capsular polysaccharides and activates CD4+ Tcells to secrete inflammatory cytokines [56]. Thus, we hypothesize that *B. fragilis* protects the host by helping it to produce inflammatory cytokines. Moreover, we found abundance increased in *P. distasonis.* A recent study suggested that *P. distasonis* may be protective against certain diseases, including multiple sclerosis, type 2 diabetes, colorectal cancer and inflammatory bowel disease. Furthermore, some reports suggested that this bacterium may even act as a potential probiotic to promote human digestive health based on microbiome or animal studies [57]. Our study demonstrated that *P. distasonis* played vital roles in maintaining the posterior intestine health of *S. schlegelii.* Another increased species, *Lysobacter* spp.*,* showed strong bacteriolytic and bacteriostatic effects, and strong antagonistic activity against a variety of pathogens [58]. The increase of this lysozyme in infected intestine suggests that after infection, these increased bacteria can destroy pathogenic bacteria and protect the health of the intestines. Taken together, changes in intestinal flora suggested that the posterior intestine quickly adjusted to the irritation of pathogenic bacteria after infection. We speculated that during the infection of *E. piscicida* in the posterior intestine of *S. schlegelii*, the intestinal microorganisms, especially for the probiotics, can be induced. The increased production of probiotics requires significant nutrient consumption, which can create overlapping ecological niches and competition for the nutrients needed for the growth of with *E. piscicida*, thus inhibiting the growth of pathogenic bacteria. At the same time, these probiotics in the posterior intestine help the host recognize bacteria, kill them and develop resistance to pathogens that enter the intestine through an inflammatory response. However, it is still necessary to study further which genes in different regions of the intestine are involved in immune protection due to the changes of these microflora.

**Figure 10.**
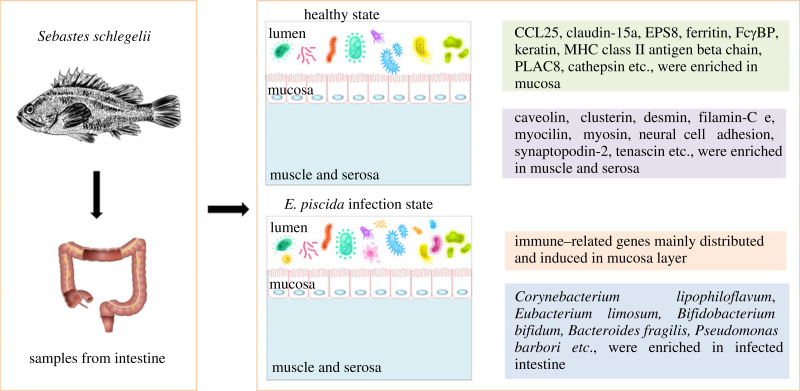
The regionally concentrated immune cells and flora in the healthy and *E. piscicida*-infected posterior intestine of *S. schlegelii*.

Here, we used ST to define how the transcriptomic landscape is spatially organized in the healthy condition and infected posterior intestine of *S. schlegelii*. Most immune-related genes were distributed in the mucosa layers of the intestine. Also, muscle and serosa layers were initially considered as mere structural support, but are also actively involved in immune response during the infection process. Immune-related genes and genes in immune-related KEGG pathways were also investigated based on spatial transcriptomes. In addition, the increasing beneficial bacteria also help the organisms fight off pathogenic infections. Meanwhile, the posterior intestine also can eliminate the harm caused by pathogenic bacteria by continuously proliferating new cells. Taken together, spatial transcriptomes, new cells and microbiota compositions have significant implications for understanding the immune mechanism that responds to *E. piscicida* infection in the posterior intestine of *S. schlegelii* (figure 10).

## 4. Material and methods

### 4.1. Bacterial challenge experiment and sampling collection

Individuals of experimental *S. schlegelii* were selected with an average body length about 15 ± 2 cm, which were purchased from Weihai, Shandong Province. Fish were cultured in a flow-through system for one week at 28°C before the infection experiment. Firstly, *E. piscicida* was cultured in LB medium at 28°C overnight with 180 rpm. Subsequently, the fish in experimental groups were immersed for 2 h in *E. piscicida* with a final concentration of 1 × 10^8^ CFU ml^−1^, while the control individuals were immersed in sterilized media (CON). Following immersion, the fish were transferred back to fresh seawater. Subsequently, posterior intestinal segments of *S. schlegelii* from CON, the infected groups were separately collected from each time point and were immediately fixed in 2.5% paraformaldehyde for TEM.

### 4.2. Tissue processing

Two replicates of posterior intestinal segment from each time point were collected, and concurrently frozen and embedded in Optimal Cutting Temperature compound (OCT, Sakura Tissue-TEK) on dry ice and stored at −80°C. Then, OCT blocks were cut with a pre-cooled cryostat at 10 µm thickness, and sections were transferred to fit the 6.5 mm^2^ oligo-barcoded capture areas on the Visium 10× Genomics slide. First, RNA quality of OCT embedded block was assessed by Agilent 2100. RNA integrity number of tissues greater than 7 were used for Visium spatial gene expression experiments. Before performing the complete protocol, the Visium Spatial Tissue Optimization Slide & Reagent kit (10× Genomics, cat. no. 1000193) was used to optimize permeabilization conditions for the tissue according to Visium Spatial Tissue Optimization User Guide (CG000238, 10× Genomics).

### 4.3. Visium experiment and sequencing

Firstly, a 10 µm frozen tissue section was placed on one of the Visium gene expression slide capture areas in a slide. After tissue H&E staining, brightfield images were acquired. Then, tissue permeabilization was performed for different minutes. Subsequently, the reverse transcription experiment was conducted, and sequencing libraries were prepared following the manufacturer's protocol using the Visium Spatial Gene Expression Slide & Reagent kit (10× Genomics, cat. no. 1000184).

### 4.4. Sequencing and data processing

Sequencing was performed with a NovaSeq S1 flow cell (Illumina) platform with an average depth of 300 million read-pairs per sample. We used in-house script to perform basic statistics of raw data, and evaluate the data quality and GC content along the sequencing cycles. Raw FASTQ files and histology images were processed by sampling with the Space Ranger (version spaceranger-1.2.0, 10× Genomics) software with default parameters. The filtered gene-spots matrix and the fiducial-aligned low-resolution image were used for down-streaming data analyses (Seurat). The Seurat package was used to perform gene expression normalization, dimensionality reduction, spot clustering and differential expression analysis. Briefly, spots were filtered for minimum detected gene count of 100 genes. Normalization across spots was performed with the SCTransform function and 3000 highly variable genes were selected for principal component analysis. For spot clustering, the first 20 PCs were used to build a graph, which was segmented with a resolution of 0.5. Wilcox algorithm was used to perform differential gene expression analysis for each cluster via FindAllMarkers function. Genes with fold change >2 and adjust *p*-value < 0.05 were defined as significantly differential expressed genes. Library construction and sequencing was performed by a commercial service provider Novogene Med NGS Clinical Laboratory (Tianjin, China).

### 4.5. Enrichment analysis

The clusterProfiler R package was used to calculate enrichment test for candidate gene sets based on hypergeometric distribution. Functional enrichment analysis was performed including GO term and KEGG pathways. Pathways with corrected *p*-value less than 0.05 were considered as significantly enriched terms.

### 4.6. DNA extraction, 16S rRNA gene amplicon processing and analysis

DNA was extracted from the slices as mentioned above. Subsequently, the V4–V5 region of the 16S rRNA gene was amplified according to the protocol with NEBNext Ultra IIDNA Library Prep Kit (Cat No. E7645). Finally, the library was sequenced on an Illumina NovaSeq platform. After data split based on barcodes and primer sequences, fastp (version 0.20.0) and Vsearch (version 2.15.0) were used to obtain the high-quality clean tags and effective tags. Subsequently, QIIME2 software (version QIIME2-202006) was used to filter, trim and merge the fastq reads to generate ASVs. Then, the annotation of species was performed based on the Silva Database. QIIME2 software was also used to perform multiple-sequence alignment and phylogenetic relationship constructions. Subsequent analysis of alpha diversities such as Observed_otus, Chao1, Shannon, Simpson, Dominance, Good's coverage and Pielou_e. and beta diversities were all performed based on the normalized data. These distance matrices were used for principal coordinates analysis to create ordinations. The R software (version 3.5.3) was used to perform the MetaStat and *t*-test analysis to investigate the different species at different taxonomic levels. In addition, the LEfSe software (version 1.0) was used to perform LEfSe analysis (LDA score threshold: 4) to capture the biomarkers. Furthermore, to investigate the functions of the communities in the samples and find out the different functions of the communities in the different groups, PICRUSt2 software (version 2.1.2-b) was used for function annotation analysis. *p*-values less than 0.05 were considered statistically significant.

## Data Availability

Additional data are provided in the electronic supplementary material [59].

## References

[1] Talbot J, Littman DR. 2021 Immune cell control of nutrient absorption. Science **371**, 1202-1203. (10.1126/science.abg6455) 33737473

[2] Soderholm AT, Pedicord VA. 2019 Intestinal epithelial cells: at the interface of the microbiota and mucosal immunity. Immunology **158**, 267-280. (10.1111/imm.13117) 31509239 PMC6856932

[3] Coombes JL, Powrie F. 2008 Dendritic cells in intestinal immune regulation. Nat. Rev. Immunol. **8**, 435-446. (10.1038/nri2335) 18500229 PMC2674208

[4] Mowat AM, Agace WW. 2014 Regional specialization within the intestinal immune system. Nat. Rev. Immunol. **14**, 667-685. (10.1038/nri3738) 25234148

[5] Mukherjee S, Hooper LV. 2015 Antimicrobial defense of the intestine. Immunity **42**, 28-39. (10.1016/j.immuni.2014.12.028) 25607457

[6] Brazil JC, Quiros M, Nusrat A, Parkos CA. 2019 Innate immune cell–epithelial crosstalk during wound repair. J. Clin. Investig. **129**, 2983-2993. (10.1172/JCI124618) 31329162 PMC6668695

[7] Kamada N, Chen GY, Inohara N, Núñez G. 2013 Control of pathogens and pathobionts by the gut microbiota. Nat. Immunol. **14**, 685-690. (10.1038/ni.2608) 23778796 PMC4083503

[8] Behringer DC, Wood CL, Krkošek M, Bushek D. 2020 Disease in fisheries and aquaculture. Mar. Dis. Ecol. **183**. (10.1093/oso/9780198821632.001.0001)

[9] Foey A, Picchietti S. 2014 Immune defences of teleost fish. Aquaculture nutrition: gut health, probiotics and prebiotics, 14-52.

[10] Salinas I, Parra D. 2015 Fish mucosal immunity: intestine//mucosal health in aquaculture, pp. 135-170. New York, NY: Academic Press.

[11] Rombout J, Van den Berg AA. 1989 Immunological importance of the second gut segment of carp. I. Uptake and processing of antigens by epithelial cells and macrophages. J. Fish Biol. **35**, 13-22. (10.1111/j.1095-8649.1989.tb03388.x)

[12] Wallace KN, Akhter S, Smith EM, Lorent K, Pack M. 2005 Intestinal growth and differentiation in zebrafish. Mech. Dev. **122**, 157-173. (10.1016/j.mod.2004.10.009) 15652704

[13] Gómez GD, Balcázar JL. 2008 A review on the interactions between gut microbiota and innate immunity of fish. FEMS Immunol. Med. Microbiol. **52**, 145-154. (10.1111/j.1574-695X.2007.00343.x) 18081845

[14] Rombout JHWM, Abelli L, Picchietti S, Scapigliati G, Kiron V. 2011 Teleost intestinal immunology. Fish Shellfish Immunol. **31**, 616-626. (10.1016/j.fsi.2010.09.001) 20832474

[15] Løkka G, Koppang EO. 2016 Antigen sampling in the fish intestine. Dev. Comp. Immunol. **64**, 138-149. (10.1016/j.dci.2016.02.014) 26872546

[16] Martin SAM, Król E. 2017 Nutrigenomics and immune function in fish: new insights from omics technologies. Dev. Comp. Immunol. **75**, 86-98. (10.1016/j.dci.2017.02.024) 28254621 PMC5495911

[17] Picchietti S, Miccoli A, Fausto AM. 2021 Gut immunity in European sea bass (*Dicentrarchus labrax*): a review. Fish Shellfish Immunol. **108**, 94-108. (10.1016/j.fsi.2020.12.001) 33285171

[18] Knoop KA, Newberry RD. 2018 Goblet cells: multifaceted players in immunity at mucosal surfaces. Mucosal Immunol. **11**, 1551-1557. (10.1038/s41385-018-0039-y) 29867079 PMC8767637

[19] Martin SAM, Dehler CE, Król E. 2016 Transcriptomic responses in the fish intestine. Dev. Comp. Immunol. **64**, 103-117. (10.1016/j.dci.2016.03.014) 26995769

[20] López Nadal A, Ikeda-Ohtsubo W, Sipkema D, Peggs D, McGurk C, Forlenza M, Wiegertjes GF, Brugman S. 2020 Feed, microbiota, and gut immunity: using the zebrafish model to understand fish health. Front. Immunol. **11**, 114. (10.3389/fimmu.2020.00114) 32117265 PMC7014991

[21] Yu Y, Wang Q, Huang Z, Ding L, Xu Z. 2020 Immunoglobulins, mucosal immunity and vaccination in teleost fish. Front. Immunol. **11**, 567941. (10.3389/fimmu.2020.567941) 33123139 PMC7566178

[22] Cao M, Yan X, Su B, Yang N, Fu Q, Xue T, Song L, Li Q, Li C. 2021 Integrated analysis of circRNA-miRNA-mRNA regulatory networks in the intestine of *Sebastes schlegelii* following Edwardsiella tarda challenge. Front. Immunol. **11**, 618687. (10.3389/fimmu.2020.618687) 33552082 PMC7857051

[23] Asp M et al. 2019 A spatiotemporal organ-wide gene expression and cell atlas of the developing human heart. Cell **179**, 1647-1660. e19. (10.1016/j.cell.2019.11.025) 31835037

[24] Ståhl PL et al. 2016 Visualization and analysis of gene expression in tissue sections by spatial transcriptomics. Science **353**, 78-82. (10.1126/science.aaf2403) 27365449

[25] Parigi SM et al. 2022 The spatial transcriptomic landscape of the healing mouse intestine following damage. Nat. Commun. **13**, 1-16. (10.1038/s41467-022-28497-0) 35149721 PMC8837647

[26] Fawkner-Corbett D et al. 2021 Spatiotemporal analysis of human intestinal development at single-cell resolution. Cell **184**, 810-826. e23. (10.1016/j.cell.2020.12.016) 33406409 PMC7864098

[27] James KR, Elmentaite R, Teichmann SA, Hold G. 2021 Redefining intestinal immunity with single-cell transcriptomics. Mucosal Immunol. **15**, 531-541. (10.1038/s41385-021-00470-y) 34848830 PMC8630196

[28] Zhang X, Cao M, Xue T, Yu H, Yang T, Yan X, Li C. 2022 Characterization of IL-17/IL-17R gene family in *Sebastes schlegelii* and their expression profiles under *Aeromonas salmonicida* and *Edwardsiella piscicida* infections. Aquaculture **551**, 737901. (10.1016/j.aquaculture.2022.737901) 37913865

[29] Rodriques SG et al. 2019 Slide-seq: a scalable technology for measuring genome-wide expression at high spatial resolution. Science **363**, 1463-1467. (10.1126/science.aaw1219) 30923225 PMC6927209

[30] Rao A, Barkley D, França GS, Yanai I. 2021 Exploring tissue architecture using spatial transcriptomics. Nature **596**, 211-220. (10.1038/s41586-021-03634-9) 34381231 PMC8475179

[31] Longo SK, Guo MG, Ji AL, Khavari PA. 2021 Integrating single-cell and spatial transcriptomics to elucidate intercellular tissue dynamics. Nat. Rev. Genet. **22**, 627-644. (10.1038/s41576-021-00370-8) 34145435 PMC9888017

[32] Lanzetti L, Rybin V, Malabarba MG, Christoforidis S, Scita G, Zerial M, Di Fiore PP. 2000 The Eps8 protein coordinates EGF receptor signalling through Rac and trafficking through Rab5. Nature **408**, 374-377. (10.1038/35042605) 11099046

[33] Chereau D, Boczkowska M, Skwarek-Maruszewska A, Fujiwara I, Hayes DB, Rebowski G, Lappalainen P, Pollard TD, Dominguez R. 2008 Leiomodin is an actin filament nucleator in muscle cells. Science **320**, 239-243. (10.1126/science.1155313) 18403713 PMC2845909

[34] Landstrom AP, Beavers DL, Wehrens XHT. 2014 The junctophilin family of proteins: from bench to bedside. Trends Mol. Med. **20**, 353-362. (10.1016/j.molmed.2014.02.004) 24636942 PMC4041816

[35] Cunningham BA, Hemperly JJ, Murray BA, Prediger EA, Brackenbury R, Edelman GM. 1987 Neural cell adhesion molecule: structure, immunoglobulin-like domains, cell surface modulation, and alternative RNA splicing. Science **236**, 799-806. (10.1126/science.3576199) 3576199

[36] Stahl PD. 1992 The mannose receptor and other macrophage lectins. Curr. Opin. Immunol. **4**, 49-52. (10.1016/0952-7915(92)90123-V) 1317711

[37] Zaidi N, Maurer A, Nieke S, Kalbacher H. 2008 Cathepsin D: a cellular roadmap. Biochem. Biophys. Res. Commun. **376**, 5-9. (10.1016/j.bbrc.2008.08.099) 18762174

[38] Zheng M, Karki R, Vogel P, Kanneganti TD. 2020 Caspase-6 is a key regulator of innate immunity, inflammasome activation, and host defense. Cell **181**, 674-687. e13. (10.1016/j.cell.2020.03.040) 32298652 PMC7425208

[39] Takeuchi O, Akira S. 2010 Pattern recognition receptors and inflammation. Cell **140**, 805-820. (10.1016/j.cell.2010.01.022) 20303872

[40] Sarma JV, Ward PA. 2011 The complement system. Cell Tissue Res. **343**, 227-235. (10.1007/s00441-010-1034-0) 20838815 PMC3097465

[41] Amatya N, Garg AV, Gaffen SL. 2017 IL-17 signaling: the yin and the yang. Trends Immunol. **38**, 310-322. (10.1016/j.it.2017.01.006) 28254169 PMC5411326

[42] Gillis C, Gouel-Chéron A, Jönsson F, Bruhns P. 2014 Contribution of human Fc*γ*Rs to disease with evidence from human polymorphisms and transgenic animal studies. Front. Immunol. **5**, 254. (10.3389/fimmu.2014.00254) 24910634 PMC4038777

[43] Brown EM, Sadarangani M, Finlay BB. 2013 The role of the immune system in governing host-microbe interactions in the intestine. Nat. Immunol. **14**, 660-667. (10.1038/ni.2611) 23778793

[44] Kayama H, Okumura R, Takeda K. 2020 Interaction between the microbiota, epithelia, and immune cells in the intestine. Annu. Rev. Immunol. **38**, 23-48. (10.1146/annurev-immunol-070119-115104) 32340570

[45] Culina S et al. 2018 Islet-reactive CD8+ T cell frequencies in the pancreas, but not in blood, distinguish type 1 diabetic patients from healthy donors. Sci. Immunol. **3**, eaao4013. (10.1126/sciimmunol.aao4013) 29429978 PMC5874133

[46] Pedersen C et al. 2018 Fecal Enterobacteriales enrichment is associated with increased in vivo intestinal permeability in humans. Physiol. Rep. **6**, e13649. (10.14814/phy2.13649) 29611319 PMC5880877

[47] Biassoni R et al. 2020 Gut microbiota in T1DM-onset pediatric patients: machine-learning algorithms to classify microorganisms as disease linked. J. Clin. Endocrinol. Metab. **105**, e3114-e3126. (10.1210/clinem/dgaa407) 32692360

[48] Mukherjee A et al. 2020 Gut microbes from the phylogenetically diverse genus *Eubacterium* and their various contributions to gut health. Gut Microbes 12, 1802866. (10.1080/19490976.2020.1802866) 32835590 PMC7524325

[49] Shirasawa Y, Shibahara-Sone H, Iino T, Ishikawa F. 2010 Bifidobacterium bifidum BF-1 suppresses Helicobacter pylori-induced genes in human epithelial cells. J. Dairy Sci. **93**, 4526-4534. (10.3168/jds.2010-3274) 20854986

[50] Chenoll E, Casinos B, Bataller E, Astals P, Echevarría J, Iglesias JR, Balbarie P, Ramon D, Genovés S. 2011 Novel probiotic *Bifidobacterium bifidum* CECT 7366 strain active against the pathogenic bacterium *Helicobacter pylori*. Appl. Environ. Microbiol. **77**, 1335-1343. (10.1128/AEM.01820-10) 21169430 PMC3067243

[51] Khailova L, Mount Patrick SK, Arganbright KM, Arganbright KM, Halpern MD, Kinouchi T, Dvorak B 2010 *Bifidobacterium bifidum* reduces apoptosis in the intestinal epithelium in necrotizing enterocolitis. Amer. J. Physiol. Gastrointest. Liver Physiol. **299**, G1118-G1127. (10.1152/ajpgi.00131.2010) 20705904 PMC2993641

[52] Fu YR, Yi ZJ, Pei J, Guan S. 2010 Effects of *Bifidobacterium bifidum* on adaptive immune senescence in aging mice. Microbiol. Immunol. **54**, 578-583. (10.1111/j.1348-0421.2010.00255.x) 21118295

[53] Mouni F, Aissi E, Hernandez J, Gorocica P, Bouquelet S, Zenteno E, Lascurain R, Garfias Y. 2009 Effect of Bifidobacterium bifidum DSM 20082 cytoplasmic fraction on human immune cells. Immunol. Investig. **38**, 104-115. (10.1080/08820130802608303) 19172489

[54] Guglielmetti S, Mora D, Gschwender M, Popp K. 2011 Randomised clinical trial: Bifidobacterium bifidum MIMBb75 significantly alleviates irritable bowel syndrome and improves quality of life-a double-blind, placebo-controlled study. Alimentary Pharmacol. Therap. **33**, 1123-1132. (10.1111/j.1365-2036.2011.04633.x) 21418261

[55] Serafini F et al. 2013 Evaluation of adhesion properties and antibacterial activities of the infant gut commensal *Bifidobacterium bifidum* PRL2010. Anaerobe **21**, 9-17. (10.1016/j.anaerobe.2013.03.003) 23523946

[56] Eribo OA, du Plessis N, Chegou NN. 2022 The Intestinal commensal, bacteroides fragilis, modulates host responses to viral infection and therapy: lessons for exploration during mycobacterium tuberculosis infection. Infect. Immunity **90**, e00321-21. (10.1128/IAI.00321-21) 34606367 PMC8788684

[57] Ezeji JC et al. 2021 Parabacteroides distasonis: intriguing aerotolerant gut anaerobe with emerging antimicrobial resistance and pathogenic and probiotic roles in human health. Gut Microbes **13**, 1922241. (10.1080/19490976.2021.1922241) 34196581 PMC8253142

[58] Li K, Hou R, Xu H, Wu G, Qian G, Wang H, Liu F. 2020 Two functional fatty acyl coenzyme A ligases affect free fatty acid metabolism to block biosynthesis of an antifungal antibiotic in *Lysobacter enzymogenes*. Appl. Environ. Microbiol. **86**, e00309-20. (10.1128/AEM.00309-20) 32144106 PMC7205486

[59] Cao M, Xue T, Huo H, Zhang X, Wang NN, Yan X, Li C. 2023 Spatial transcriptomics and microbiota reveal immune mechanism that response to pathogen infection in the posterior intestine of *Sebastes schlegelii*. *Figshare*. (10.6084/m9.figshare.c.6416957) PMC9944294 36974664

